# Development of the multi-epitope chimeric antigen rqTSA-25 from *Taenia saginata* for serological diagnosis of bovine cysticercosis

**DOI:** 10.1371/journal.pntd.0006371

**Published:** 2018-04-12

**Authors:** Rafaella P. M. Guimarães-Peixoto, Paulo S. A. Pinto, Marcus R. Santos, Tiago J. Zilch, Paula F. Apolinário, Abelardo Silva-Júnior

**Affiliations:** 1 Laboratório de Inspeção de Produtos de Origem Animal, Departamento de Veterinária, Universidade Federal de Viçosa, Viçosa, Minas Gerais, Brazil; 2 Laboratório de Virologia Animal, Departamento de Veterinária, Universidade Federal de Viçosa, Viçosa, Minas Gerais, Brazil; University of Texas Health Science Center, UNITED STATES

## Abstract

Bovine cysticercosis is a worldwide distributed zoonosis caused by the larval form of *Taenia saginata* present in bovine muscles. The diagnosis is based on the postmortem inspection at slaughterhouses and consists of the macroscopic visualization of lesions caused by cysticercosis in muscle sites. However, parasitized animals can pass unnoticed during sanitary inspection. Thus, the objective of this study was to characterize and evaluate the performance of different peptides from different regions of *T*. *saginata* for the cysticercosis diagnosis using enzyme-linked immunosorbent assay. We generated and evaluated a new recombinant protein chimera derived from the fusion of different peptides. We selected three distinct regions of *T*. *saginata* and predicted six peptides with antigenic potential (EP2–EP7). These peptides were analyzed individually and selected for generating a new chimeric recombinant protein. The new protein was termed rqTSA-25, and its performance rates were: 93.3% sensitivity (confidence interval (CI) = 76–98%), 95.3% specificity (CI = 82–99%), 93% positive predictive value (CI = 76–98%), 95% negative predictive value (CI = 82–99%), and 95% accuracy. In the immunoblot, this protein showed no false positive or false negative reaction. Thus, the use of rqTSA-25 is recommended for the diagnosis of bovine cysticercosis.

## Author summary

*Taenia saginata* cysticercosis occurs worldwide. The prevalence related is higher mainly in developing countries, causing damage to public health and economic losses. Through our study, a new antigen with high diagnostic potential was developed for use in laboratory serological tests, aiming at the detection of bovine cysticercosis. In this way, it was possible to contribute significantly to the improvement of the diagnosis of this disease.

## Introduction

Bovine cysticercosis is one of the major public and animal health problems worldwide, and particularly in Brazil where it causes economic losses for slaughterhouses and farmers [1]. The human being is the definitive host of the bovine cysticercosis agent, *Taenia saginata* [2], while the cattle act as intermediate hosts, contaminating themselves directly or indirectly ingesting human feces containing eggs of *T*. *saginata* [3].

Several authors reported the need to investigate and implement the enzyme-linked immunosorbent assay (ELISA) serological test as a tool for meat inspection, seeking greater efficiency in the diagnosis of bovine cysticercosis [3–6]. The European Food Safety Authority (EFSA) also recommended the development and validation of a serodiagnostic test for bovine cysticercosis routine diagnosis [7].

The main performance parameters (sensitivity and specificity) of the ELISA for animal cysticercosis diagnosis have been reported by several authors [3,8–11]. However, satisfactory results have not yet been obtained when naturally infected animals were the diagnostic target, because of the insufficient amount of circulating antibodies [12,13]. Thus, it is necessary to search for antigenic alternatives, which may contribute to the improvement of the ELISA. Bioinformatics techniques may be useful for the selection of proteins with high antigenicity in order to optimize immunological tests.

The production and application of recombinant proteins and chimeras have been used in the diagnosis of different diseases such as *Toxoplasma gondii* [14] and *Leishmania infantum* [15] infection, classical swine fever [16], and also *Taenia solium* infection, mainly focusing on human neurocysticercosis [17] but not on the diagnosis of animal cysticercosis. In the diagnosis of bovine cysticercosis, antigenic proteins of *Taenia crassiceps* larvae, especially those with low molecular mass, have been used in its immunological diagnosis [18, 19].

In this study, we aimed to investigate target proteins with a great affinity for the antibodies produced by the host under different immunological conditions in order to improve the serological tests. We constructed, characterized, and evaluated a new recombinant chimeric protein based on different peptides from the target molecular regions of the TSA18, TSA16, and Tsp36 proteins of *T*. *saginata*, investigating its potential in the diagnosis of bovine cysticercosis compared to the protocols already developed with the *T*. *crassiceps* larva antigens.

## Methods

### Computational characterization

The selection of B lymphocyte epitopes was performed using the primary sequences and the three-dimensional structures of the 18-kDa oncosphere proteins, TSA16, and Tsp36. The primary sequences were retrieved from GenBank (accession no. ADO86979.1, AFU50753.1, and ID Q7YZT0.1, respectively). The three-dimensional structure of Tsp36 was obtained from the Protein Data Bank (PDB, ID 2BOL) [8]. The structural model of TSA16 was constructed by homology from its amino acid sequence using the Protein Fold Recognition Server (PHYRE2) [20]. The stereochemical and energetic quality of the model was evaluated using the Ramachandran plot of Probity [21] and ProSA-web [22]. The programs used for the mapping of linear epitopes from the primary sequence of the proteins were: BepiPred [23], ABCPred [24], AAPPred [25], and Elipro [26] (from the three-dimensional sequence). The resulting data converged in the epitope described below (Table 1), which was synthesized by Genscript (Piscataway, NJ, USA) with > 95% purity.

**Table 1:** Description of *Taenia saginata* proteins, peptide nomenclature, and amino acid sequence.

Description of proteins	Peptide	Amino acid sequence
18 kDa oncosphere antigen [*T*. *saginata*]	EP1	Guimarães-Peixoto et al. (2016)
Antigen TSA16 [*T*. *saginata*]	EP2*	CSGDTSLRSCMHWSHKG
	EP3*	CVRHVSVSASPVSKPHH
	EP4*	CGRILLQGLLANTEYVL
Antigen TSA36 [*T*. *saginata*] Heat shock protein 20 homolog [*T*. *saginata*]	EP5*	CSIFPTRDSRDLSSRRR
	EP6*	CIQPREFHPELEYTQPG
	EP7*	CSEVQERQLAVKNKEGL

* A Cysteine residue was added to the peptides to facilitate adsorption on the enzyme-linked immunosorbent assay (ELISA) plates

### Design of synthetic gene and production of recombinant protein

A multi-epitope synthetic gene was designed. Three coding sequences from antigenic peptides were joined, resulting in rqTSA-25. A flexible linker (GGGS)^2^ (Registry of Standard Biological Parts, accession no. BBa_K1486003, http://parts.igem.org/Part:BBa_K1486003) and a hard linker were added between the different peptides (EAAAKEAAAK) [27]. A 6xHis-tag coding sequence was added upstream of the stop codon of each synthetic gene for affinity purification of recombinant proteins. The sequence was codon-optimized for *Escherichia coli* expression. The gene was synthesized by Genscript and the synthetic genes were cloned into the pET29a(+) expression vector. The recombinant plasmid was used to transform *E*. *coli* BL21-CodonPlus (DE3)-RIL (Stratagene, La Jolla, California, USA) strain, and protein expression was performed by inoculation of an overnight culture in Luria Bertani medium containing 50 μg/mL kanamycin. The culture was diluted (1:100) and incubated in TB (Terrific Broth; Sigma Chemical Co., St Louis, MO, USA) with shaking (180 rpm) at 37°C to an optical density (OD) of 0.8 at 600 nm. The culture was induced with 0.5 mM isopropyl β-D-1-thiogalactopyranoside (IPTG) for 4 h at 180 rpm at 30°C. Cells were lysed using a sonicator (Ultrasonic liquid processors S-4000-010; Misonix Inc., Farmingdale, NY, USA), and soluble and insoluble protein fractions were analyzed by 15% sodium dodecyl sulfate-polyacrylamide gel electrophoresis (SDS-PAGE). Soluble fractions of the recombinant protein were affinity purified by Fast Protein Liquid chromatography (FPLC), using 1-mL His-Trap-FF crude column (GE Healthcare Bio-Sciences AB, São Paulo, Brazil).

### Serum samples

#### Samples for peptide-based ELISA

The bovine serum samples used were previously stored in Laboratório de Inspeção de Produtos de Origem Animal, Departamento de Veterinária, Universidade Federal de Viçosa. The samples of bovine serum used for peptides analysis were divided into four distinct groups as follows: Group 1 (G1, n = 30) was composed of blood samples of bovine experimentally infected with *T*. *saginata* eggs [10] and submitted to necropsy; Group 2 (G2, n = 30) consisted of samples collected from naturally infected animals whose cysticercosis diagnosis was performed after routine postmortem; Group 3 (G3, n = 30) was composed of samples from livestock slaughtered in commercial slaughterhouses that were negative for cysticercosis and other diseases after routine postmortem inspection; and Group 4 (G4, n = 15) consisted of samples from livestock that tested negative for cysticercosis but were positive for other diseases such as tuberculosis (n = 2), hydatidosis (n = 4), and fasciolosis (n = 9). All animal groups were examined by Brazilian methodology of meat inspection.

#### Samples for rqTSA25-based ELISA

In the determination of the number of samples for the performance analysis of the rqTSA-25 protein (S1 Flowchart), the following samples were used: G1 (n = 30), G2 (n = 30), G3 (n = 30), and G4 (n = 15) were used in the parallel analysis of *T*. *crassiceps* antigen.

#### Samples for immunoblot by rqTSA25

In order to perform the immunoblot, the samples were selected as follows: Group 1 (n = 15), composed of samples of experimentally infected animals; Group 2 (n = 15), composed of samples of naturally infected animals; Group 3 (n = 15), composed of samples of animals negative to cysticercosis after standard postmortem examination; Group 4 (n = 3), composed of samples of cysticercosis-negative animals that were reared in isolation; and Group 5, composed of samples of animals with tuberculosis (n = 2), fasciolosis (n = 4), and hydatidosis (n = 4) observed after *post mortem* examination (S2 Flowchart).

### Serological tests

#### Synthetic peptide- and rqTSA25-based ELISA

The optimal dilutions for the reagents used in the ELISA assays were determined by block titration. The peptides were diluted with 0.5 M (1 μg/mL) carbonate-bicarbonate buffer, pH 9.4, and incubated overnight at 4°C. After three washes with 0.15 M saline solution, pH 7.4, containing 0.05% Tween 20, the plates were blocked with 1% Molico (Nestlé, Araçatuba, São Paulo, Brazil) denatured powdered milk in phosphate-buffered saline (PBS) and incubated for 1 h at 37°C. The livestock serum samples were diluted 1:100 in the blocking solution with 0.05% Tween 20 for 1 h at 37°C. After three washes, a rabbit anti-bovine IgG antibody conjugated to peroxidase (A5295, Sigma Chemical Co.) was added to the plates at a 1:10,000 dilution, followed by incubation and washing steps as described above. The spectrophotometric detection was initiated with an incubation with a solution of 0.1% o-phenylenediamine dihydrochloride (OPD) (P-8287, Sigma Chemical Co.) and 0.003% H_2_O_2_ in 0.2 M citrate-phosphate buffer, pH 5.0, for 30 min at 37°C. The reaction was stopped with H_2_SO_4_ (4N), and the plates were read by a spectrophotometer (ELx800 Biotek Instruments, Winooski, Vermont, USA) at a 492 nm wavelength. All reagents were added to the plates at a volume of 100 μL/well, except the blocking solution (200 μL/well).

#### *T*. *crassiceps* antigen-based ELISA

*T*. *crassiceps* larval antigens were obtained via intraperitoneal inoculation of BALB/c female mice [28]. After collection, the cysticerci were immediately frozen (−20°C). Afterwards, the cysticerci were lyophilized at 25°C, homogenized, and mixed with 0.15 M saline solution, resulting in a final concentration of 6.5 to 10%. The diluted cysticerci were homogenized in ice using a tissue homogenizer (Potter) (Wheaton, Millvile, New Jersey, USA) and centrifuged at 17,400 × *g* for 30 min at 4°C. A protease inhibitor (PMSF, P-7626, 0.25 M–10 μL/mL; Sigma Chemical Co.) was added to the supernatant, and the antigen was stored (−20°C) until used.

The polystyrene plates (Thermo Fisher Scientific, Waltham, Massachusetts, USA) were sensitized with the diluted antigens (40 μg/mL) in a 0.5-M buffered solution of carbonate-bicarbonate, pH 9.6. The ELISA plates were coated for 1 h at 37°C. After three washes in saline solution containing 0.05% Tween 20, the reactive sites were blocked using 5% denatured milk in PBS, pH 7.4, for 1 h at 37°C. After additional three washes, the samples were diluted in 1% denatured milk in PBS, pH 7.4. The plates were incubated for 30 min at 37°C. After washing, rabbit anti-bovine IgG antibody conjugated to peroxidase (1:5.000) was added to each well, and the incubation and wash procedures were repeated. The chemiluminescent reaction was initiated using a solution of 0.1% OPD and 0.003% H_2_O_2_ in 0.2 M citrate-phosphate buffer, pH 5.0. After a 5-min incubation, the reaction was stopped using H_2_SO_4_ (4N), and the plates were read by a spectrophotometer at a wavelength of 492 nm. All reagents were added at a volume of 100 μL/well, except the blocking solution (200 μL/well).

#### Determination of antigen performance

The reactivity of the peptides was compared to the results obtained from the ELISA tests. Each ELISA reaction was performed in triplicate, and the mean ODs were calculated. The OD values were normalized to a standard reference plate, and the correction factor was calculated [29]. The cut-off point was selected based on the mean OD plus two standard deviations, obtained from analyses of negative serum control samples collected from livestock raised in isolation, kept under controlled conditions, and slaughtered under rigorous inspection.

To determine the performance of the antigens in the ELISA test, the following criteria were used: determination of sensitivity (G2) and specificity (G3–G4), considering all samples of these groups. The positive and negative predictive values and accuracy were calculated [30–31]. To test the performance rates, a CI of 95% was taken into account.

#### Immunoblot

To assess the potential of rqTSA-25 antigen in an alternative serological test, rqTSA-25 was used in immunoblot assays. rqTSA-25 was used at the concentration of 8 μg per channel and separated according to its molecular mass by 15% SDS-PAGE along with the broad range molecular marker (161–0317; BioRad, Hercules, California, USA). The protein was transferred from the gel to a 0.45-μm nitrocellulose membrane (Millipore Corp., Billerica, Massachusetts, USA) as described by Towbin et al. (1979). The transfer was performed for 2 h at 4°C, with a fixed amperage of 250 mA. After transfer, the membrane was stained with 0.05% Ponceau S in double distilled water for qualitative visualization of the transfer. The membranes were separated into 3-mm wide strips and washed with 0.05% Tween 20 in saline (0.15 M NaCl) and subjected to immunodiagnosis. The strips had their remaining reactive sites blocked with 5% skimmed milk powder in Tris-saline, pH 7.4, for 1 h. Excess of blocking solution was removed. Serum samples were added at a dilution of 1:100 in 1% skimmed milk powder in Tris-saline, pH 7.4, for 1 h. The strips were washed with 0.05% Tween 20 in Tris-saline, pH 7.4, for six times for 5 min each. The rabbit anti-bovine IgG antibody conjugated to peroxidase was added to the 1:10.000 dilution for 1 h, followed by repeated washing procedures. Reactions were revealed by chromogen solution (diaminobenzidine 50 mg/15 mL, 0.15% H_2_O_2_ in PBS, pH 7.4) until the reactive bands of the serum control samples were observed. The reaction was stopped with distilled water. The reagents were added in 1-mL volume per channel. The whole test was performed on an oscillating table with constant and slow stirring at 25°C.

### Ethics committee approval

The norms of conduct for the use of animals in research from the Ethics Committee for Animal Experimentation of the Federal University of Viçosa were followed according to the Process reference 20/2011 CEUA/UFV.

## Results

### Evaluation of the three-dimensional model of TSA16 protein and the selected peptides

**Fig 1:**
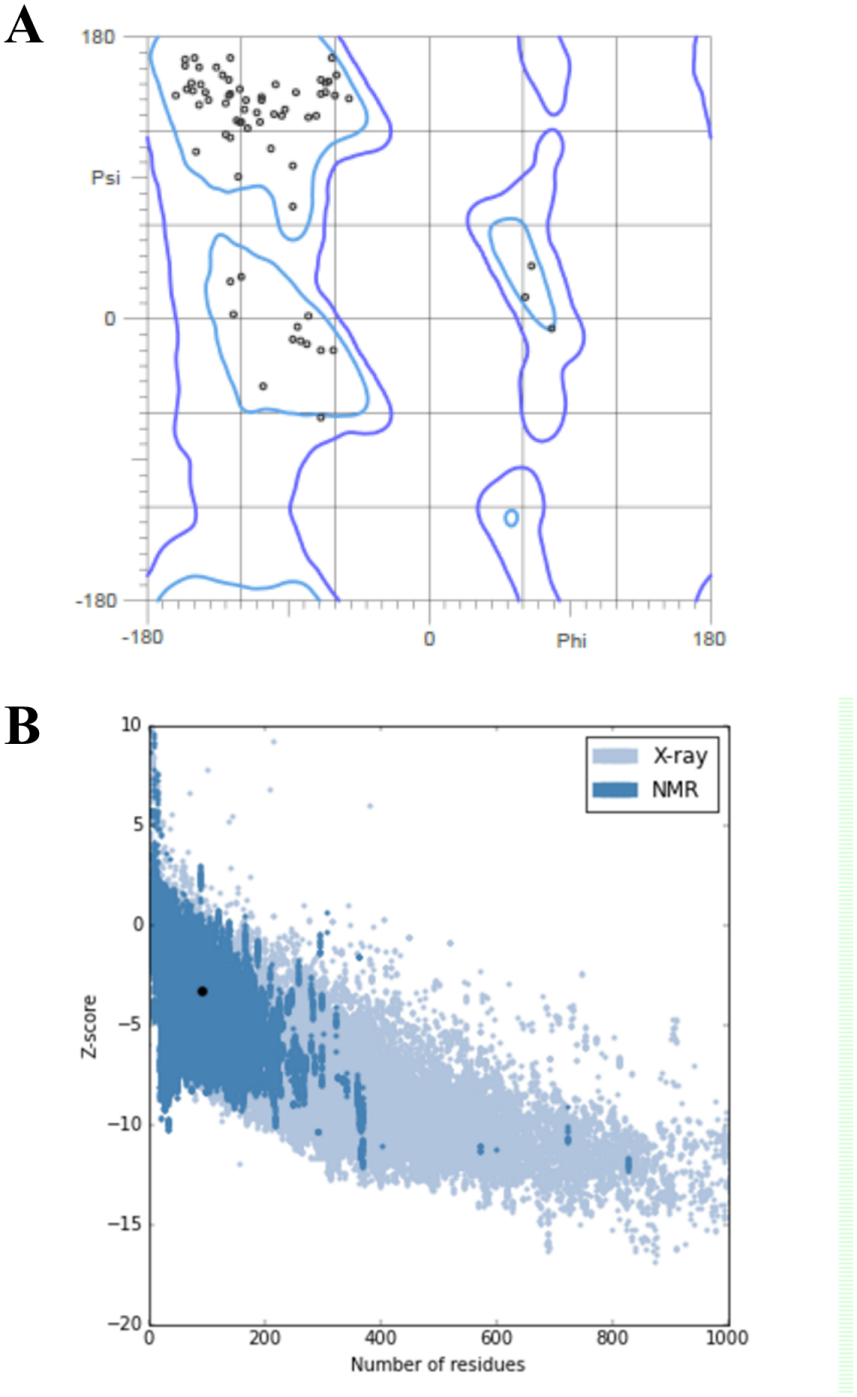
*In vitro* validation of the TSA16 three-dimensional model. A) Map of Ramachandran. The plot shows that 93.5% of the residues are in favorable regions and 97.8% in the permitted regions. B) Overall evaluation of the model using ProSA web. The punctuation of the modeled structure (highlighted point) is in the range with the scores found on proteins of similar size using nuclear magnetic resonance (NMR).

For the homology modeling of TSA16, the protein sequence deposited in the PDB (ID 4JMG) was used as a template, and a model with 97.8% of the residues in regions allowed stereochemically was generated (Ramachandran plot, Probity analysis) (Fig 1A). The analysis of the global structure of the model showed a structure compatible with the structural studies using X-ray and NMR (Fig 1B), resulting in a z-score of −3.28.

**Fig 2:**
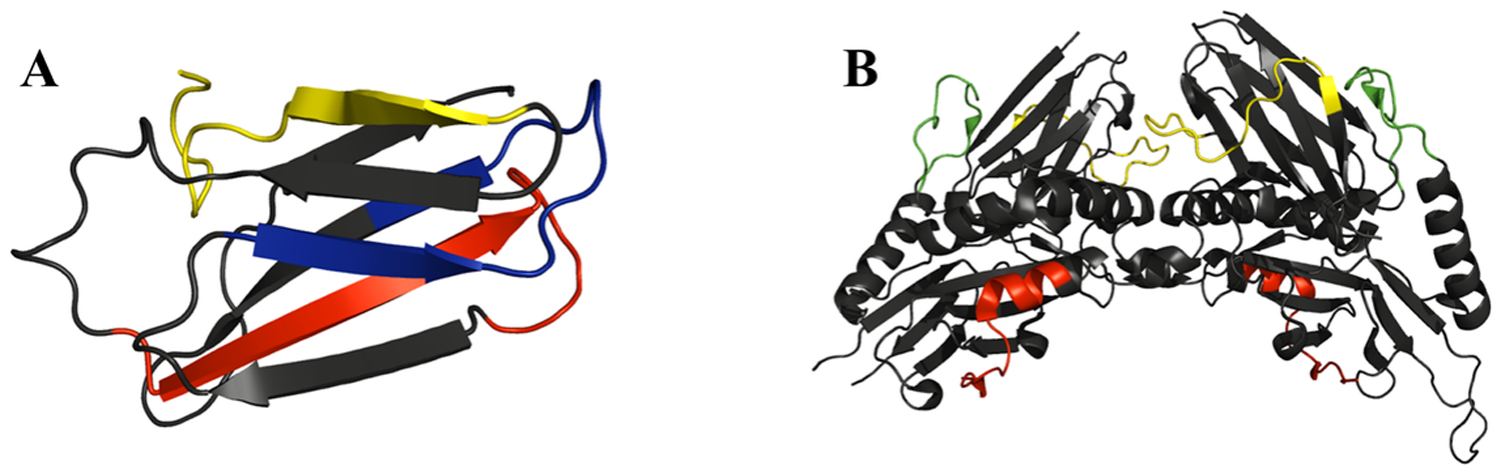
A) Three-dimensional model of TSA16; EP2 in light yellow, EP3 in red, and EP4 in blue. B) Three-dimensional structure of Tsp36 (Protein Data Bank (PDB) ID 2BOL); EP5 in red, EP6 in green, and EP7 in yellow.

From the prediction of the peptides, it was possible to select antigenic regions in several secondary structures of the evaluated proteins. The peptides EP2, EP3, EP4, and EP7 were mostly exposed to the solvent in the beta sheets and loop regions, whereas EP5 and EP6 were present in alpha-helix and loop regions (Fig 2).

The peptides with diagnostic potential were discriminated through their ELISA-antigen-antibody interaction performances based on sensitivity, specificity, positive predictive value (PPV), negative predictive value (NPV), and accuracy (ACC) as described in Table 2.

**Table 2:** Performance rates *(%) of *Taenia saginata* peptides in the diagnosis of bovine cysticercosis by enzyme-linked immunosorbent assay (ELISA).

Performance (%)	Peptides					
	EP2	EP3	EP4	EP5	EP6	EP7
Sensibility	70 (50–84)	83 (64–93)	66 (47–82)	83 (64–93)	80 (60–91)	46 (28–65)
Specificity	60 (44–73)	75 (60–86)	57 (42–72)	42 (27–57)	57 (42–72)	75 (60–86)
Positive predictive value	53 (37–69)	69 (51–83)	51 (35–67)	49 (34–63)	55 (40–70)	56 (35–74)
Negative predictive value	75 (57–87)	87 (71–95)	72 (54–85)	79 (57–92)	81 (62–92)	68 (53–80)
Accuracy	64	79	61	59	67	64

*Confidence interval of 95%

Considering the performance analysis of each peptide per control serum group, we highlighted the behavior of EP3 and EP5, which showed a satisfactory differentiation between the positive and negative samples, especially when the samples of bovines with natural infection were analyzed. Both peptides identified 83% of the positive samples, a main factor in the selection of a serological test as the cattle have a low concentration of circulating antibodies (Table 3). The EP3 peptide had the greatest capacity to identify negative samples (83%).

**Table 3:** Percentages of positivity (G1, G2) or negativity (G3, G4) for bovine cysticercosis of the samples submitted to the enzyme-linked immunosorbent assay (ELISA) for each peptide.

Serum group	Peptides					
	EP2	EP3	EP4	EP5	EP6	EP7
G1	50	63	53	86	60	73
G2	70	83	66	83	80	46
G3	73	83	70	46	50	80
G4	33	60	33	33	73	66

Group 1: samples of experimentally infected animals; Group 2: samples of naturally infected animals; Group 3: animals negative to cysticercosis after standard postmortem examination; Group 4: samples of cysticercosis-negative animals that were reared in isolation and cysticercosis-negative animals that presented other diseases (tuberculosis, fasciolosis, and hydatidosis) after routine postmortem examination

In this study, only linear epitopes wee predicted based on convergent regions between the software and regions not described in other studies. Therefore, after analyzing the ELISA performance, two predicted peptides (EP3 and EP5) stood out as they obtained values of sensitivity and/or specificity superior to the other peptides. They were selected to be part of a novel recombinant chimeric protein. The new protein, called rqTSA-25, is a combination of the EP1 peptide that was previously characterized by our group [18] with the peptides EP3 and EP5.

**Fig 3:**
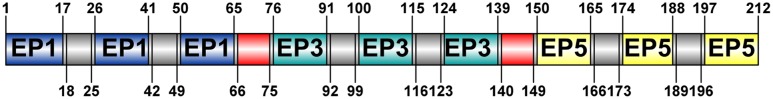
rqTSA-25 amino acid sequence. Predicted amino acid sequences of rqTSA-25, showing the flexible linker in gray and hard linker in red.

The rqTSA-25 (Fig 3) was successfully produced, expressed, and purified from the gene constructed on the basis of peptide selection. This new protein was used as a new antigen in the standardization and evaluation of the ELISA test, showing its good performance for the serological diagnosis of bovine cysticercosis.

### Performance of rqTSA-25

**Fig 4:**
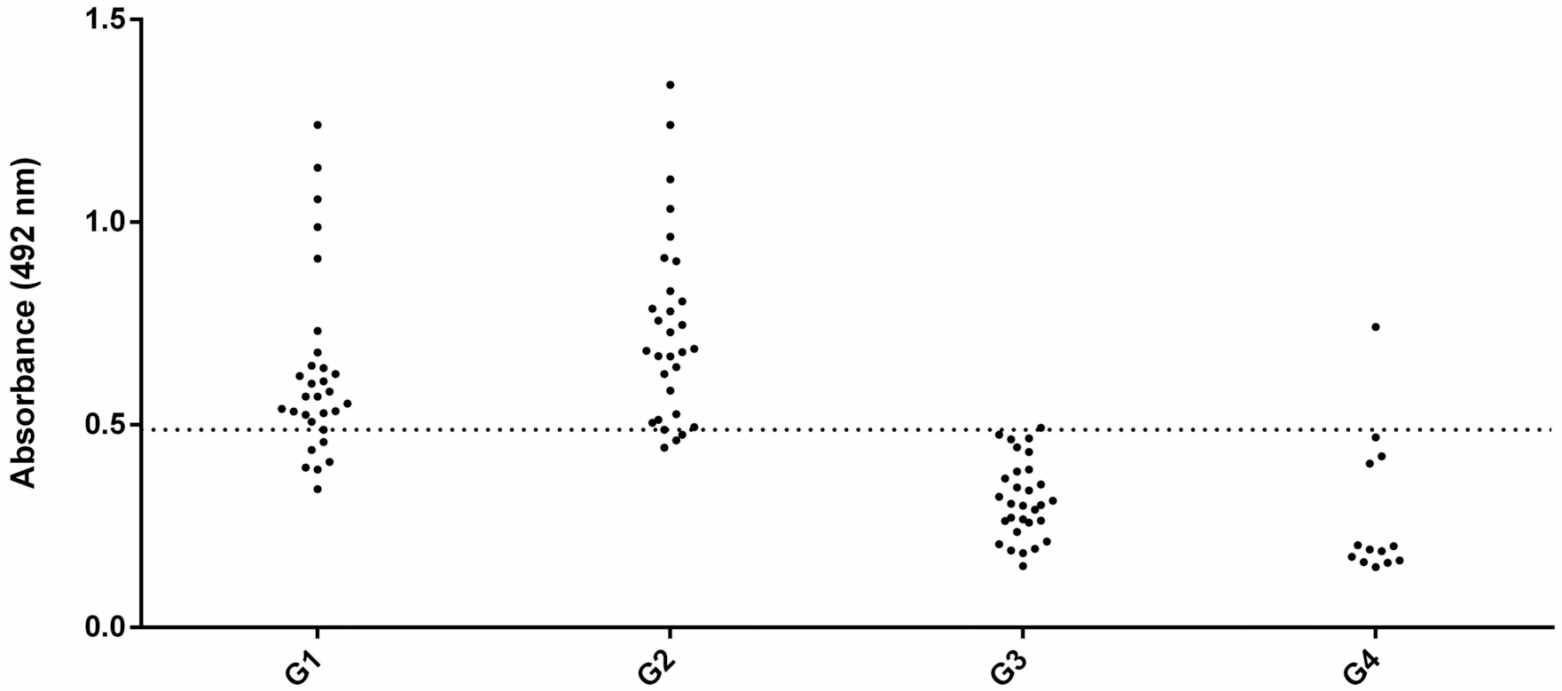
Reactivity to the enzyme-linked immunosorbent assay (ELISA) using rqTSA-25 (cut off = 0.488). G1- blood samples from bovines from an estate with no history of cysticercosis, which were experimentally infected; G2- samples of naturally infected animals whose diagnosis of cysticercosis was obtained after postmortem inspection conducted in routine supervised slaughterhouses by the official inspection service; G3- negative bovine samples for cysticercosis and other diseases slaughtered under the same conditions as the animals in G2; G4—bovine samples negative for cysticercosis but presenting other diseases (tuberculosis, hydatidosis, and fasciolosis) diagnosed at slaughter under the same conditions as the animals in G2.

rqTSA-25 showed the following results in the diagnostic performance of bovine cysticercosis: 93.3% sensitivity (confidence interval (CI) = 76–98%), 95.3% specificity (CI = 82–99%), 93% PPV (CI = 76–98%), 95% NPV (CI = 82–99%), and 95% accuracy (Fig 4). Compared to the heterologous antigen of *T*. *crassiceps*, the following ELISA performance parameters were obtained: 70% sensitivity (CI = 56–80%), 82% specificity (CI = 73–89%), 72% PPV (CI = 60–84%), 80% NPV (CI = 70–87%), and 78% accuracy [20].

### Detection of anti-rq-TSA25 antibodies by immunoblot

**Fig 5:**
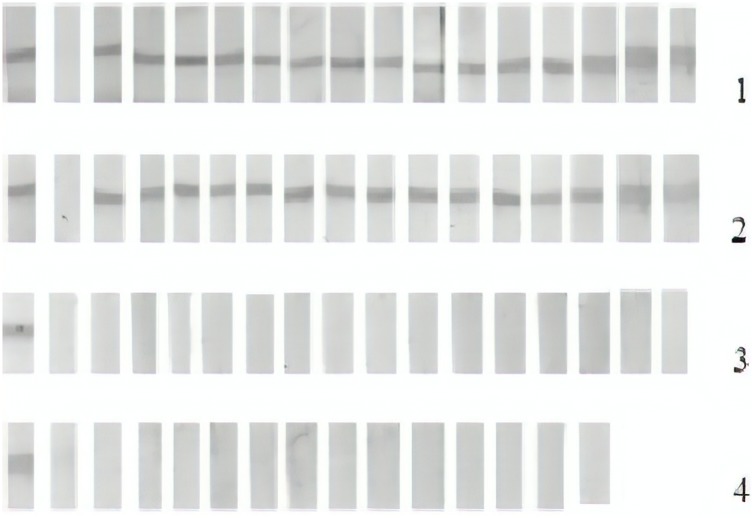
Reactivity of the samples to the immunoblot. Sample positive control (first column) and negative control (second column), and samples from other groups of control sera (columns 3 to 17). Line/Group 1, experimentally infected bovine serum samples; Line/Group 2, naturally infected bovine serum samples; Line/Group 3, bovine serum samples negative for cysticercosis during routine inspection; Line/Group 4, negative samples to bovine cysticercosis during routine inspection but positive to other pathologies (tuberculosis, fasciolosis, or hydatidosis.

To confirm the efficacy of rqTSA-25 in other serological tests, some samples (n = 59) from different serum control groups were analyzed by immunoblot, a confirmatory diagnostic test with a higher specificity than that of the ELISA test. No false positive or false negative reactions were observed in this test (Fig 5).

## Discussion

The *T*. *saginata* protein currently used in the diagnosis of bovine cysticercosis is already immunologically characterized as an 18-kDa protein called HP6 [32–34] or TSA18 [35,36], which is an adhesion protein of the cysticercus oncosphere and has been used in the diagnosis of animal and human cysticercosis [37]. Two other proteins are also reported for diagnostic purposes: the 16-kDa protein TSA16 and the Tsp36 protein, an important protein in the chaperon group [38]. However, the *T*. *solium* proteins homologous to TSA16 and Tsp36, named Tso16 and Tso36, respectively, present an antigenicity already characterized, indicating a possible diagnostic application similar to the corresponding *T*. *saginata* proteins [39–41].

The sensitivity and specificity reported for peptides from the 16-kDa protein were: EP2, 70% (CI = 50–84%) and 60% (CI = 44–73%), respectively; EP3, 83% (CI = 64–93%) and 75% (CI = 60–86%), respectively, and EP4, 66% (CI = 47–82%) and 57% (CI = 42–72%), respectively. The EP3 peptide had the best performance as it showed better rates and maintained a greater balance between sensitivity and specificity compared to the other peptides, reflecting a higher overall test performance, with an accuracy of 79%. Probably this is related to the localization of the 16-kDa protein, which is present in the activated oncosphere of the parasite as there is evidence of a secretory signal in this group of antigens [42], indicating that the 16-kDa antigen is associated with the secretory vesicles produced by activated oncosphere of cysticercus, associated with parasite-host interaction [39,43–45]. In addition, after the recognition of the parasite by the host immune system, specific antibodies are produced to combat the infection, as also indicated by the serological detection of cysticercosis by the ELISA test in this study.

EP5–EP7 peptides were obtained from the 36-kDa heat-shock protein. This protein is related to the ability of the parasite to develop response to heat-shock of the host since, the heat-shock response is a general homeostatic mechanism that protects cells and organisms from the deleterious effects of the environment in which they are [46,47]. During the stages of development of the parasite, sudden changes in temperature can occur, and adaptation to these conditions is essential for the survival of the parasite and its effective transmission [48]. The sensitivity and specificity values reached by the peptides belonging to this group were: EP5, 83% (CI = 64–93%) and 42% (CI = 27–57%), respectively, EP6, 80% (CI = 60–91%) and 57% (CI = 42–72%), respectively, and EP7, 46% (CI = 28–65%) and 75% (CI = 60–86%), respectively. Thus, the performance of peptides EP3 and EP7, which were able to recognize the absence of anti-cysticercus antibodies at high rates, was outstanding.

For the analysis of cross-reaction between bovine cysticercosis and other diseases, samples belonging to G4 (EP3 and EP6), which indicated fewer cross-reactions compared to the control serum group, were analyzed. For EP3, there were six false-positive reactions (1 tuberculosis, 4 fasciolosis, and 1 hydatidosis), and for EP6, four false-positive reactions were detected (2 tuberculosis, 1 fasciolosis, and 1 hydatidosis).

The use of chimeric proteins constituted by peptides derived from epitope regions has been attempted, aiming at the improvement of diagnostic techniques. Some studies have shown higher performance values of these proteins in comparison to isolated peptides [49,50]. rqTSA-25 confirmed those results, having greater sensitivity and specificity than the selected peptides.

Compared to the heterologous antigen (*T*. *crassiceps*, Tcra), the rqTSA-25 performance values were higher, indicating a higher antigen-antibody affinity and proving to be an important ally in improving the diagnostic test. rqTSA-25 could recognize 96.6% of the naturally infected animal samples, whereas Tcra recognized 70% of the samples, suggesting a major advantage of using rqTSA-25 in field disease recognition. Other advantages of using rqTSA-25 may be its superior performance in naturally infected animals and in mono-cysticercosis cattle diagnosis, as these animals generally have a low amount of circulating antibodies. Notably, mono-cysticercosis carcasses represent the majority of cases of cysticercosis in slaughterhouses [51].

rqTSA-25 specificity was 95.3%, while that of Tcra was 82%, indicating that rqTSA-25 resulted in few non-specific and cross-reactive ELISA reactions. Considering the reactions of serum samples from G3 (cysticercosis-negative samples) and G4 (samples negative for cysticercosis but with other diseases) with rqTSA-25, there was only one non-specific reaction in both cases, accounting for 3.33% (1/30) and 7.7% (1/13), respectively, whereas Tcra showed 11.6% (7/60) of these reactions in G3 and 28.5% (8/28) in G4.

The high specificity value favors the use of rqTSA-25 as an antigen for the serological diagnosis of bovine cysticercosis, especially in the confirmatory assays of suspect cases, when using previous diagnostic criteria for screening by other methodologies results in high sensitivity and low specificity. In addition, no false positive or false negative reaction in the samples analyzed by immunoblot test was observed.

It is known that even with a great homology between the species of tapeworm, there will always be a greater affinity in the antigen-antibody interaction in the specific parasite-host relationship, therefore, modern technologies such as the production of recombinant antigens, to approach the maximum of naturally occurring conditions in the animal during the different stages of infection.

The cost of implementing a more sensitive and specific tool for the diagnosis of bovine cysticercosis should be related to the benefits of a reduced risk of human infection (teniasis), impacting on the potential of transmission of bovine cysticercosis and also benefiting the livestock sector, reducing the economic losses [6].

Currently, studies on the use of recombinant antigens for the diagnosis of bovine cysticercosis are scarce. Most of the research remains focused on the diagnosis of *T*. *solium* as it is the parasite that causes swine and human cysticercosis, with a greater impact on public health [52–54]. However, considering the economic and health importance, and the occurrence of bovine cysticercosis and its diffusion in different world regions, it is important to improve the research on bovine cysticercosis and its diagnosis and, consequently, control.

## Conclusion

When evaluating the new rqTSA-25 recombinant antigen, the performance rates of the ELISA test for the diagnosis of bovine cysticercosis were better than those of other currently developed laboratory protocols. The results proved that there was an effective differentiation between positive and negative samples, highlighting the high discriminatory potential of rqTSA-25 in natural infection diagnosis, contributing to the routine diagnosis in slaughterhouses and the epidemiological researches in the livestock sector.

## Supporting information

S1 Foreign Language Abstract Abstract in Portuguese (Brazil). (DOCX)

S1 Checklist STARD checklist. (DOCX)

S1 Flowchart ELISA flow diagram. (PDF)

S2 Flowchart IMMUNOBLOT flow diagram. (PDF)

S1 Materials Materials transfer office. (PDF)
