# Supplementary material for: Development of the multi-epitope chimeric antigen rqTSA-25 from Taenia saginata for serological diagnosis of bovine cysticercosis
Source: PLoS Negl Trop Dis. 2018 Apr 12;12(4):e0006371. doi: 10.1371/journal.pntd.0006371 (PMC6078323; doi:10.1371/journal.pntd.0006371)
Supplement: S1 Foreign Language Abstract — (DOCX) [file pntd.0006371.s001.docx]

**Resumo**

A cisticercose bovina é uma zoonose de distribuição mundial ocasionada pela forma larvar da *Taenia saginata* quando presente na musculature de bovinos. O diagnóstico é realizado durante inspeção post mortem em matadouros-frigoríficos, consistindo de visualização macroscópica das lesões causadas pelos cisticercos em regiões musculares pré definidas. Entretanto, animais parasitados podem passer despercebidos durante a inspeção sanitaria de rotina. Dessa forma, o objetivo desse estudo foi caracterizar e verificar a performance de diferentes peptídeos oriundos de diferentes regiões de *T. saginata* para o diagnóstico da cisticercose utilizando um teste imunoenzimático (ELISA). Foi desenvolvida e testada uma nova proteína quimera recombinante derivada da fusão de diferentes peptídeos. Foram selecionadas três regiões distintas de *T. saginata* e preditos seis peptídeos com potencial antigênico (EP2–EP7). Esses peptídeos foram analisados individualmente e selecionados para a construção de uma nova proteína quimera recombinante. A nova proteína foi denominada de rqTSA-25, e seu desempenho foi: 93,3% sensibilidade (interval de confiança (IC) = 76–98%), 95,3% especificidade (IC = 82–99%), 93% valor preditivo positivo (IC = 76–98%), 95% valor preditivo negativo (IC = 82–99%), e 95% de acurácia. No immunoblot, a proteína não mostrou nenhuma reação falso positiva ou falso negativa. Dessa forma, a utilização da rqTSA-25 é recomendada para o diagnóstico sorológico da cisticercose bovina.
