# Supplementary figures and images for: Development of the multi-epitope chimeric antigen rqTSA-25 from Taenia saginata for serological diagnosis of bovine cysticercosis
Source: PLoS Negl Trop Dis. 2018 Apr 12;12(4):e0006371. doi: 10.1371/journal.pntd.0006371 (PMC6078323; doi:10.1371/journal.pntd.0006371)

Prototypical STARD diagram to report flow of participants through the study

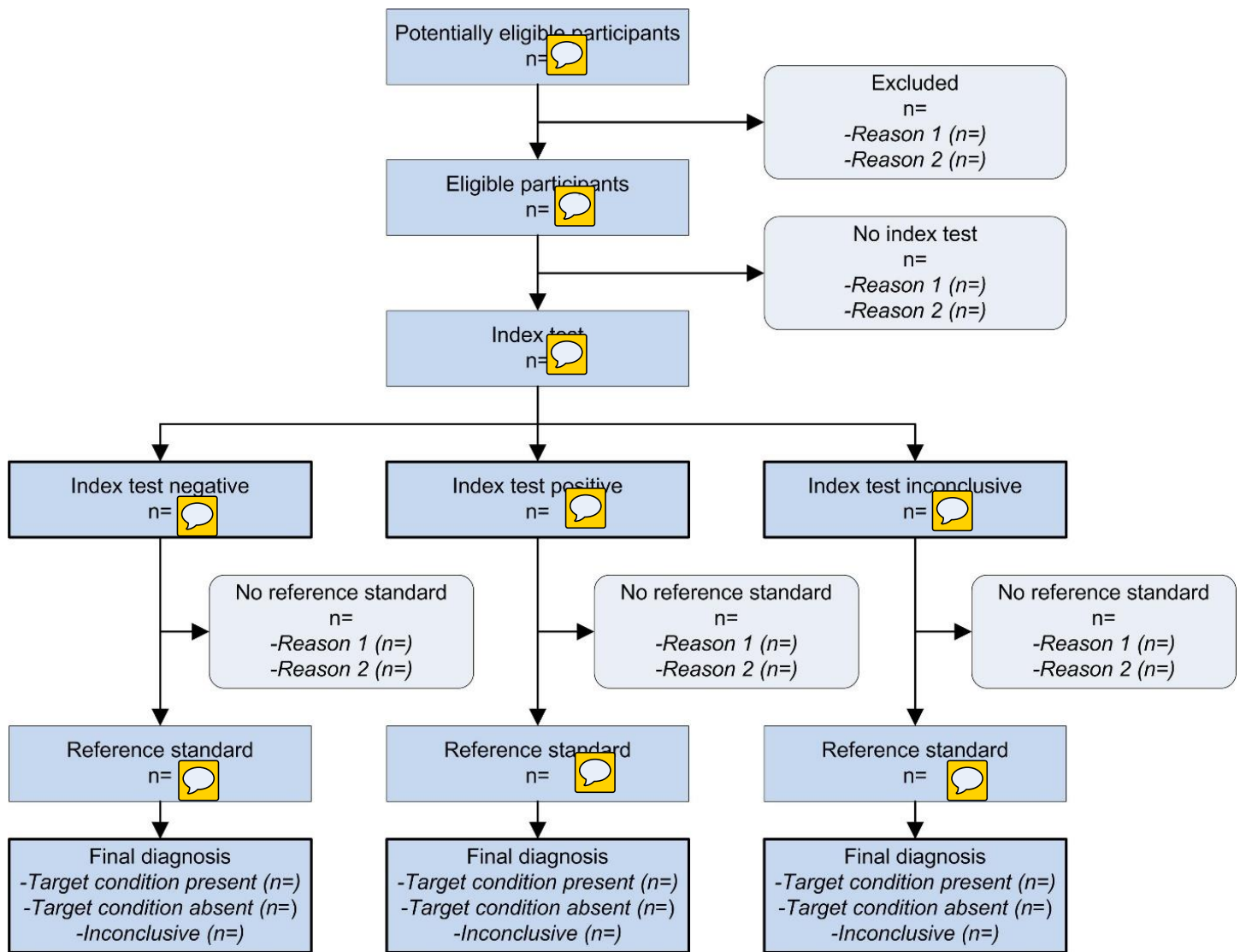

Supplement: S1 Flowchart — (PDF) [file pntd.0006371.s003.pdf]

Prototypical STARD diagram to report flow of participants through the study

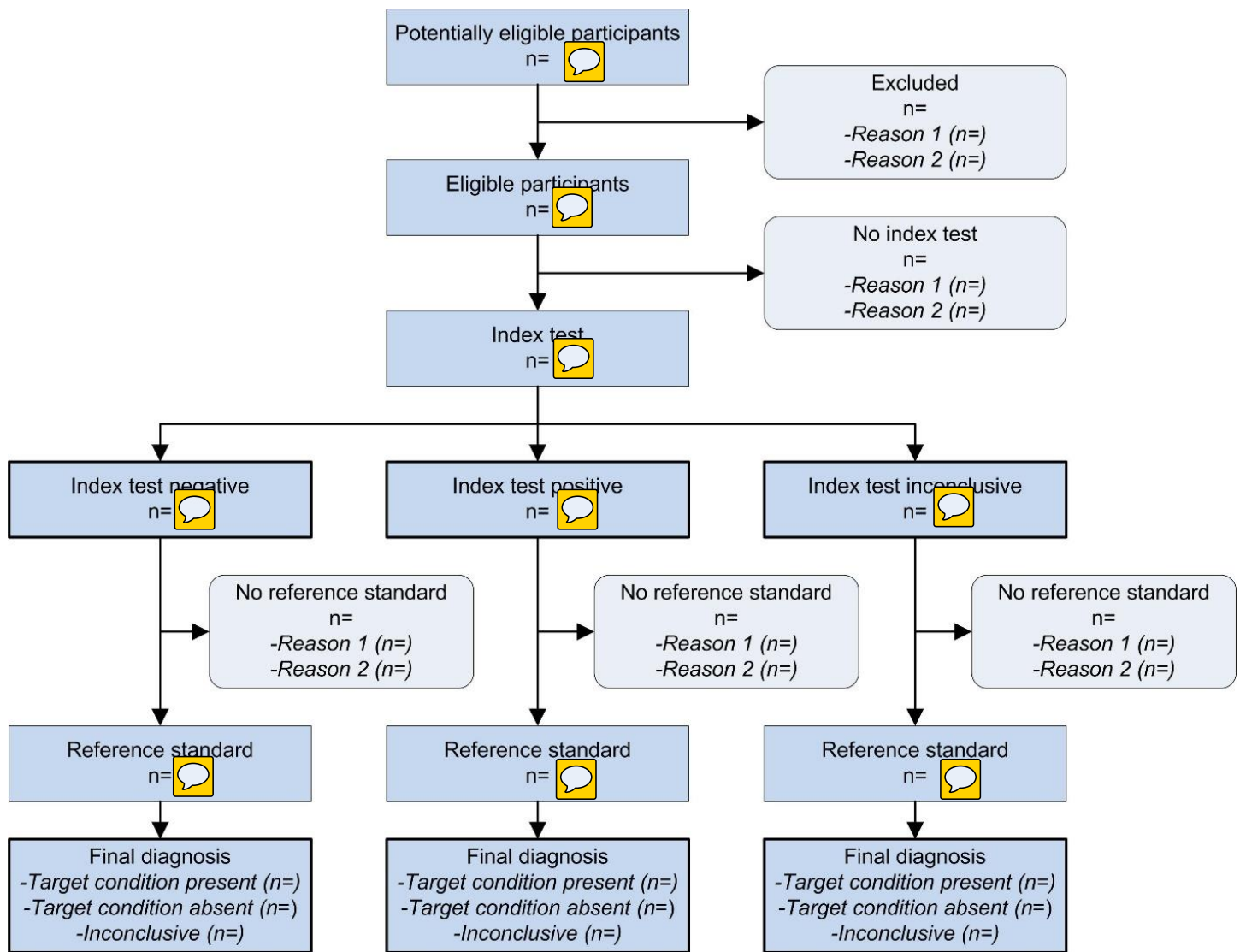

Supplement: S2 Flowchart — (PDF) [file pntd.0006371.s004.pdf]
