# Supplementary material for: Development of the multi-epitope chimeric antigen rqTSA-25 from Taenia saginata for serological diagnosis of bovine cysticercosis
Source: PLoS Negl Trop Dis. 2018 Apr 12;12(4):e0006371. doi: 10.1371/journal.pntd.0006371 (PMC6078323; doi:10.1371/journal.pntd.0006371)
Supplement: S1 Materials — (PDF) [file pntd.0006371.s005.pdf]

Materials Transfer Office

The authors declare that all the data underlying the manuscript “Development of the multi-epitope chimeric antigen rqTSA-25 from *Taenia saginata* for serological diagnosis of bovine cysticercosis”, are available for the online data conference of the library of the Federal University of Viçosa, Minas Gerais, Brazil and the entire patent application process is deposited with INPI (National Institute of Prority Industrial) under the number BR 10 2017 016571 0.
